# Investigation of ANN architecture for predicting shear strength of fiber reinforcement bars concrete beams

**DOI:** 10.1371/journal.pone.0247391

**Published:** 2021-04-02

**Authors:** Quang Hung Nguyen, Hai-Bang Ly, Thuy-Anh Nguyen, Viet-Hung Phan, Long Khanh Nguyen, Van Quan Tran

**Affiliations:** 1 Thuyloi University, Hanoi, Vietnam; 2 University of Transport Technology, Hanoi, Vietnam; 3 University of Transport and Communications, Hanoi, Vietnam; RMIT University, AUSTRALIA

## Abstract

In this paper, an extensive simulation program is conducted to find out the optimal ANN model to predict the shear strength of fiber-reinforced polymer (FRP) concrete beams containing both flexural and shear reinforcements. For acquiring this purpose, an experimental database containing 125 samples is collected from the literature and used to find the best architecture of ANN. In this database, the input variables consist of 9 inputs, such as the ratio of the beam width, the effective depth, the shear span to the effective depth, the compressive strength of concrete, the longitudinal FRP reinforcement ratio, the modulus of elasticity of longitudinal FRP reinforcement, the FRP shear reinforcement ratio, the tensile strength of FRP shear reinforcement, the modulus of elasticity of FRP shear reinforcement. Thereafter, the selection of the appropriate architecture of ANN model is performed and evaluated by common statistical measurements. The results show that the optimal ANN model is a highly efficient predictor of the shear strength of FRP concrete beams with a maximum R^2^ value of 0.9634 on the training part and an R^2^ of 0.9577 on the testing part, using the best architecture. In addition, a sensitivity analysis using the optimal ANN model over 500 Monte Carlo simulations is performed to interpret the influence of reinforcement type on the stability and accuracy of ANN model in predicting shear strength. The results of this investigation could facilitate and enhance the use of ANN model in different real-world problems in the field of civil engineering.

## 1. Introduction

In aggressive environments, the load-bearing capacity of reinforcing bars in the concrete structure can be seriously declined due to steel corrosion. Accordingly, the performance of reinforced concrete structures could be reduced [1–3]. To prevent this phenomenon, numerous solutions have been proposed to avoid steel corrosion in reinforced concrete structures, such as increase the concrete cover layer to protect the reinforcements, the use of high-performance concrete (HPC), or waterproof paint [4]. However, these solutions lead to an increase in cost structures. Some investigations have shown that the corrosion resistance of reinforced concrete could be increased by using fiber-reinforced polymer (FRP) bars to replace traditional steel [3, 5–7]. Various types of FRP could be used, such as non-metallic glass fiber-reinforced polymers (GFRP), carbon fiber-reinforced polymer (CFRP), basalt fiber-reinforced polymer (BFRP), or aramid fiber-reinforced polymer (AFRP). Indeed, FRP bars have many advantages, such as good mechanical properties, small self-weight, easy transportation, installation, non-conductive, non-magnetic, and low heat conductivity [3, 8–12]. Therefore, FRP bars have been proposed as longitudinal and shear reinforcements for different types of concrete structures exposed to various aggressive environments, mostly sewage water, seawater…[9, 13–15]. Numerous experimental investigations have shown that the general flexural theory of reinforced concrete structures is also valid for FRP reinforced concrete beams [16]. However, the material properties of FRP are significantly different compared with that of steel reinforcement. Especially, the elastic modulus of FRP is lower than that of steel reinforcement, so that the shear behaviors of FRP bar-reinforced concrete beams such as shear strength, deformation, and crack width are different from those of traditional steel reinforcement concrete [17, 18]. Moreover, the FRP bars have only linear elastic behavior without any plasticity until failure [19]. In addition, the general flexural theory of reinforced concrete structures is moderately applied for concrete beams using FRP as flexural reinforcement [20, 21], but using FRP as shear reinforcement raise the complexity of mechanism behavior of concrete beams. Therefore, it is difficult to apply the existing shear strength prediction models of reinforced concrete beams to estimate FRP reinforced concrete beams.

Until now, many theoretical and experimental studies have been carried out to predict the shear strength of FRP reinforced concrete beams [3, 16, 22–25]. Most investigations have tried to come up with simple predictive equations based on different shear mechanisms to facilitate the FRP bars design in concrete structures [2, 26]. However, the accuracy of these predictive equations seems to be limited. In fact, these equations are empirically developed by different experimental results, where each one is performed for a specific case study, such as varying the geometrical variables of beams or investigating the feasibility of using one specific type of reinforcement. Therefore, these equations could hardly be universal for predicting the shear strength of different FRP reinforced concrete beams [27]. In addition, numerous design standards of FRP reinforced concrete beams have been introduced to determine the shear strength, namely ACI 440.1R-06 [28], CNR-DT 203/2006 [29], CSA S806-12 [30], CSA S6-14 [31], and JSCE (Japan Association for Civil Engineering, 1997). However, several previous studies [16, 23, 26] have shown that these design guidelines are often too conservative for shear strength estimation of FRP concrete beams. Thus, the designed amount of FRP bars are often more considerable than the actual amount. The overestimation induces to increase cost structures [23]. Therefore, the development of an efficient and universal model to increase the shear strength prediction accuracy of FRP concrete beams is crucial.

Over the last four decades, artificial intelligence (AI) or machine learning (ML) is gradually becoming popular and applied in many technical fields [32–34]. The artificial neural network (ANN), a well-known ML algorithm, has been widely used in the construction field [35–37]. Many complex problems related to civil engineering, such as structural engineering [38, 39], material sciences [40–43], geotechnical engineering [44–48], and earth sciences [49–53] have been favorably resolved by applying ANN. Using machine learning techniques, the shear strength prediction of FRP concrete beams has also been the subject of some investigations in the literature. Nehdi et al. [2] have used a genetic algorithm approach and 168 experimental results to propose shear strength design equations for FRP reinforced concrete beams. In the investigation of Abbasloo et al. [54], a rule-based method has been used to predict the shear strength of FRP reinforced concrete without shear reinforcement. The authors have collected 176 experimental results from the literature and show the robustness of the machine learning approach against the shear strength design equations in both aspects of accuracy and reliability. These investigations indicate that machine learning algorithms are powerful numerical tools that can resolve numerous complex relationships between different components and optimize them to acquire the targeted mechanical properties, such as the shear strength of FRP reinforced concrete. Therefore, the primary purpose of this investigation is to propose an efficient model to increase the shear strength prediction accuracy of FRP concrete beams.

In this work, the performance of ANN model is investigated to predict the shear strength of FRP reinforced concrete. Although the most effective machine learning algorithm is ANN model, its performance depends strongly on the selection of ANN architecture. Therefore, this investigation simultaneously performs the determination and optimization of the ANN architecture for better prediction of the shear strength of FRP reinforced concrete beams. To achieve this goal, numerous experimental data from the literature are collected and randomly divided into two parts: the training part (70% of data) and the testing part (30% of data). Moreover, Monte Carlo simulations (MCS) are also performed to verify the convergence and feasibility of the proposed model. Thanks to Monte Carlo simulations, a minimal number of simulations that ensure the reliability of prediction results are determined. The best ANN architecture is derived and used to predict the shear strength of FRP reinforced concrete beams with the help of three statistical measurements, including the Coefficient of Determination (R^2^), Mean Absolute Error (MAE), and Root Mean Square Error (RMSE). Finally, the prediction capability of the best ANN architecture is investigated in function of different types of FRP reinforcements.

## 2. Database construction

In this study, 125 experimental results have been collected from 15 experimental works [16, 55–68] to construct the database (Table 1). Summary and source information for the database are also summarized, including the amount of data and the corresponding percentage. It is worth noticing that all the research works include FRP concrete beams with both flexural and shear reinforcement.

**Table 1:** Summary of the data and references used in this study.

References	Number of data	Percentage
Ahmed et al. [55]	3	2.4
Shehata [68]	6	4.8
Alseyed et al. [56]	3	2.4
Nakamura and Higai [57]	7	6.4
Tottori and Wakul [58]	34	27.2
Nagasaka et al. [59]	24	16
Vijay and Kumar [60]	4	3.2
Maruyama and Zhao [61]	9	7.2
Zhao et al. [62]	5	4
Maruyama and Zhao [63]	4	4
Duranovic et al. [64]	2	1.6
Shehata et al. [16]	2	3.2
T. Okamoto et al. [65]	11	8.8
Alkhrdaji et al. [66]	4	3.2
Niewels [67]	7	5.6
**Total**	**125**	**100**

Based on the above database, each sample consists of a vector of dimension 1 × 9, including three groups of inputs. The first group consists of beams’ characteristics, such as beam width, effective depth, the ratio of the shear span to the effective depth, and compressive strength of concrete. The second group consists of flexural reinforcement parameters such as longitudinal FRP reinforcement ratio and modulus of elasticity of longitudinal FRP reinforcement. The last group consists of shear reinforcement parameters, namely the FRP shear reinforcement ratio, the tensile strength of FRP shear reinforcement, and modulus of elasticity of FRP shear reinforcement. The output vector is of dimension 1 × 1 and consists of the value of the shear strength of FRP beams with flexural and shear reinforcement. Statistical information of the input and output variables used in this study are presented in Table 2, including the mean values, along with the minimum, maximum, standard deviation (StD), median, skewness values (StD).

**Table 2:** Summary of the input and output variables of beams used in this study.

Parameters	Sym.	Unit	Min	Median	Mean	Max	StD*	SK**
Beam width	b_w_	mm	135.00	200.00	202.02	300.00	46.44	0.34
Effective depth	d	mm	230.00	253.00	306.21	600.00	86.32	1.70
The ratio of the shear span to the effective depth	a/d	-	1.19	2.69	2.67	4.31	0.72	-0.10
Compressive strength of concrete	f_c_	MPa	23.00	35.30	37.07	71.60	8.13	1.11
Longitudinal FRP reinforcement ratio	ρ_f_	%	0.53	1.71	1.74	4.65	0.94	1.19
Modulus of elasticity of longitudinal FRP reinforcement	E_f_	10^3^ MPa	29.00	94.00	102.39	206.00	58.01	0.69
FRP shear reinforcement ratio	ρ_s_	%	0.04	0.40	0.52	1.50	0.43	1.04
Tensile strength of FRP shear reinforcement	f_s_	MPa	322.00	903.00	958.69	2040.00	390.71	0.74
Modulus of elasticity of FRP shear reinforcement	E_s_	10^3^ MPa	30.00	58.00	72.97	144.00	36.75	0.38
Shear strength	V	kN	49.00	150.10	176.94	536.00	99.56	0.84

*StD = Standard deviation

**SK = Skewness.

**Fig 1:**
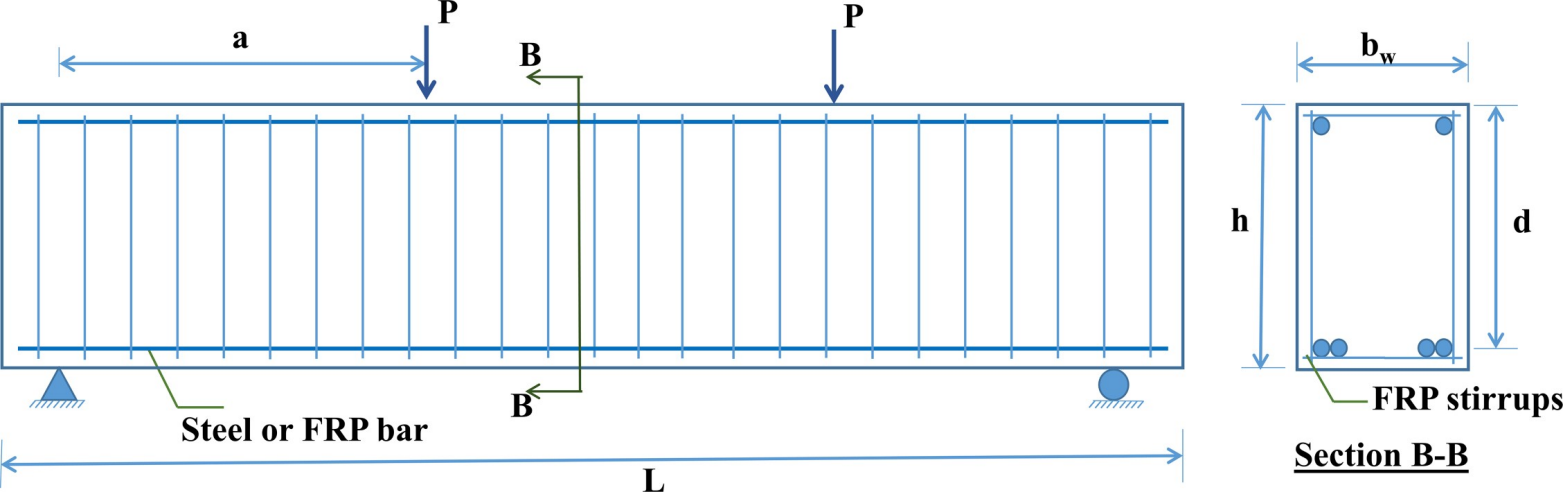
Experimental diagram of FRP beams with FRP stirrups and longitudinal reinforcement.

In the presented database, concrete beams with four types of FRP reinforcement are used, namely carbon fiber reinforced polymer (CFRP), glass fiber reinforced polymers (GFRP), aramid fiber-reinforced polymer (AFRP), and vinyl fiber-reinforced polymer (VFRP). Besides, 27 samples also use steel as flexural reinforcement. A summary of the reinforcement type used in this study is presented in Table 3 in separating samples with flexural reinforcement, shear reinforcement, and a combination of flexural reinforcement and shear reinforcement. Finally, the beam test diagram is illustrated in Fig 1.

**Table 3:** Summary of the reinforcement type used in this study.

Reinforcement type	Samples	Percentage (%)
**Flexural reinforcement**		
Steel	27	21.6
AFRP	41	32.8
CFRP	38	30.4
GFRP	19	15.2
**Shear reinforcement**		
VFRP	10	8.0
AFRP	20	16.0
CFRP	53	42.4
GFRP	42	33.6
**Flexural reinforcement + Shear reinforcement**		
Steel + VFRP	10	8.0
Steel + AFRP	2	1.6
Steel + CFRP	7	5.6
Steel + GFRP	8	6.4
AFRP + VFRP	0	0
AFRP + AFRP	16	12.8
AFRP + CFRP	23	18.4
AFRP + GFRP	2	1.6
CFRP + VFRP	0	0
CFRP + AFRP	2	1.6
CFRP + CFRP	23	18.4
CFRP + GFRP	13	10.4
GFRP + VFRP	0	0
GFRP + AFRP	0	0
GFRP + CFRP	0	0
GFRP + GFRP	19	15.2

## 3. Methods

### 3.1. Artificial neural network

**Fig 2:**
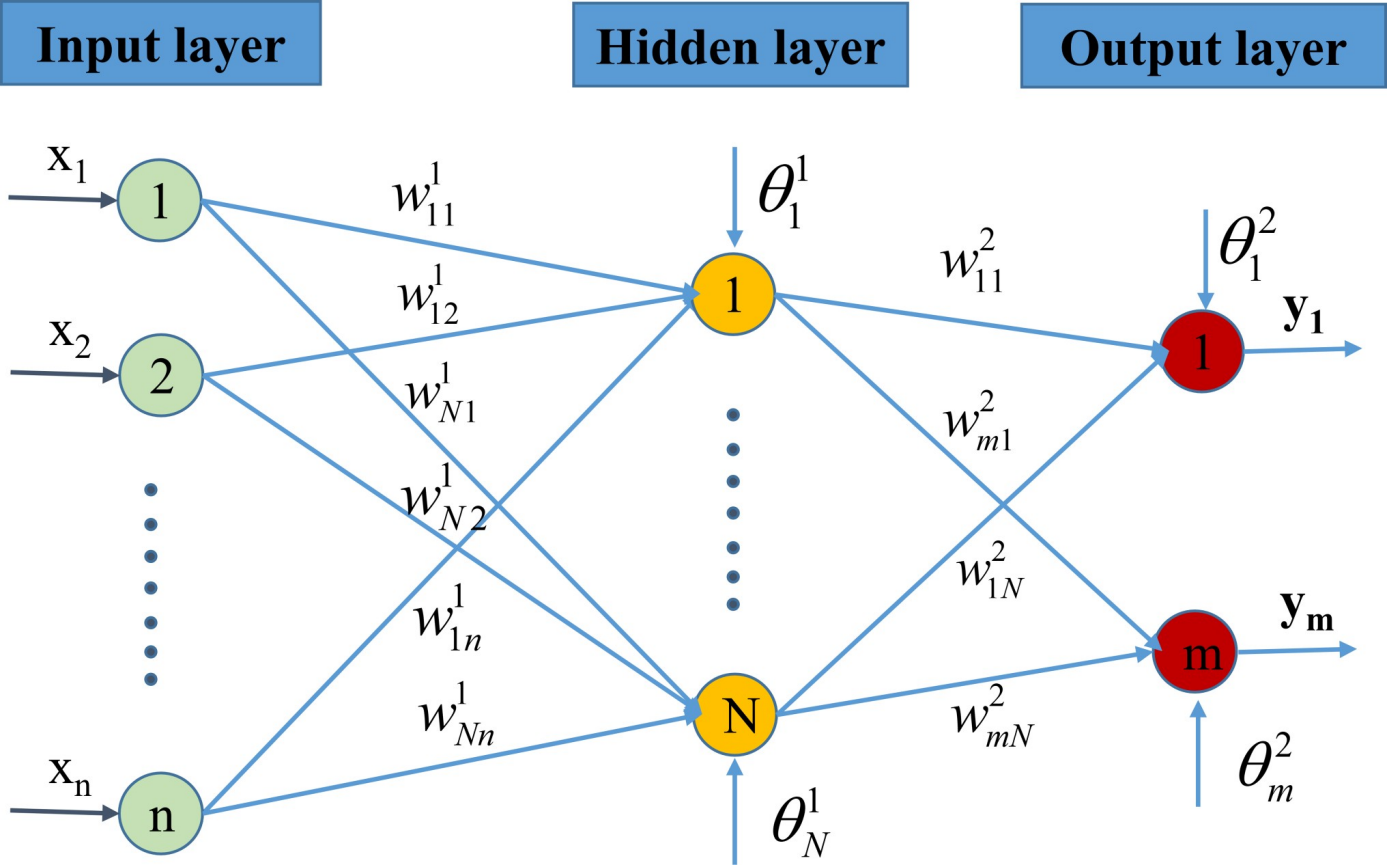
Artificial neural network model.

An artificial neural network (ANN) is an information processing model simulated according to the information processing way of biological neuron systems. It is made up of a large number of neurons that are interconnected through connections to solve a particular problem. The ANN network structure is made up of three or more layers, depicted in Fig 2, consisting of (i) an input layer that is the leftmost layer of the network, representing the input parameters, (ii) an output layer is the rightmost layer of the network, representing the results achieved, and (iii) one or more hidden layers denoting the logical inference of the network. The input layer contains the information x_i_ (i = 1,2,.., n) from the original data. The values x_i_ are then multiplied by a weighted value $w_{jk}^{1}$ (j = 1,2,…, N) where k = 1,2,.., n. The value $\overset{n}{\underset{k=1}{\Sigma }}w_{jk}^{1}x_{k}+\theta_{j}^{1}$ becomes the input of the hidden layer, and continues to be multiplied by another weighted value $w_{ij}^{2}$ (i = 1,2,…, m), becoming the input of the output layer. The values $\theta_{j}^{1}$ represent the bias associated with each neuron in the hidden layer.

Finally, the output value is obtained as $\overset{L}{\underset{j=1}{\Sigma }}w_{ij}^{2}\left(\overset{n}{\underset{k=1}{\Sigma }}w_{jk}^{1}x_{k}+\theta_{j}^{1}\right)+\theta_{i}^{2}$.

The learning process of the neural network corresponds the process in which the network continuously changes the values $w_{jk}^{1}$ and $w_{ij}^{2}$ in order to change the output value *y*_*i*_, until the *y*_*i*_ value meets the requirements (to reach a certain error compared with the target output). The back-propagation algorithm (BPNN) is widely used to train the neural network [69]. The BP algorithm is a slope reduction technique that minimizes errors for a particular training pattern in adjusting the weight by a small amount each time (or each epoch, iteration). Nerve cells in each layer are linked to the front and rear nerve cells with each associated weight, as presented above. However, the standard backpropagation algorithm typically has a slow convergence rate [70]. One of the generalization-type methods, such as the Bayesian Regularization (BR) [71, 72] is commonly used to avoid such a convergence problem and obtain a lower mean square error between the output of ANN and the target [73].

Bayesian regularization is a network training function that updates the weight and bias values according to Levenberg-Marquardt optimization. It minimizes a combination of squared errors and weights, then determines the correct combination to produce a network with high prediction accuracy. From a Bayesian point of view, the regularization corresponds to a prior probability distribution over free parameters *w* of the model. Using the notation of MacKay [71], the regularized target function can be written as:

$$F(w)=\beta E_{D}(w)+\alpha E_{W}(w)$$ (1)

where *E*_*D*_ is the sum of squared errors, *E*_*w*_ is the sum of square of the network weights, *α* and *β* are target function parameters. In ANN algorithm, the weights are considered random variables, and therefore the density function is written according to Baye’s rules [74] as follow:

$$P\left(w|D,\alpha ,B,M\right)=\frac{P\left(w|D,\beta ,M\right)P\left(w|\alpha ,M\right)}{P\left(D|\alpha ,\beta ,M\right)}$$ (2)

where *w* is the vector of network weights, *D* is the data vector, and *M* is the neural network model. The optimization of regularization parameters *α* and *β* demands for solving the Hessian matrix of *F(w)* at the minimum point *wMP*. This technique can reduce the potential to arrive at a local minimum, thus increasing the network’s generalizability.

### 3.2. Monte Carlo simulation

Numerical prediction models involving Monte Carlo simulation can explain the output results’ variation through statistical analysis. The Monte Carlo method is potent to calculate the influence of the input variability on the output results using numerical AI models [40, 44, 75, 76]. In this study, the objective of the Monte Carlo method is to randomly repeat simulations, taking into account the variability in the input space, then calculate the corresponding output through a machine learning model [77]. The robustness of Monte Carlo simulation and sensitivity of input variables can be evaluated through statistical performance criteria of the output results. The statistical convergence of Monte Carlo simulation has been carried out using the normalized convergence criteria as follow [78–80]:

$$\mathrm{Normalized}\;\mathrm{Conv}.(Y)=\frac{1}{\underline{Y}}\frac{1}{S}\sum_{i=1}^{S}Y_{i}$$ (3)

where *Y* is the mean value of the considered variable *Y*, and *S* is the number of Monte Carlo simulations. For statistical analysis, the convergence function provides efficient information of the computational time and reliability of the predicted results.

### 3.3. Performance criteria

To evaluate the effectiveness of the proposed ANN model, several evaluation criteria are proposed, including mean absolute error (MAE), root mean square error (RMSE), and the coefficient of determination (R^2^). Precisely, MAE represents the average amplitude of the model error but does not indicate the biased trend of the model output and actual values. When MAE = 0, the model value completely coincides with the actual value, thus the model could be considered as "ideal". The values of MAE are in the range of (0; +∞). Besides, root mean square error (RMSE) is one of the fundamental criteria and is commonly used to evaluate the predictive modeling results. It is common to use the RMSE to denote the mean magnitude of the error. In particular, RMSE is very sensitive to large error values. Therefore, the closer the RMSE is to the MAE, the more stable the model error. Criteria MAE, RMSE do not indicate the deviation between the model’s output value and the actual value, and in the range of (0; +∞). R^2^ is the coefficient of determination that represents the suitability of the data with the algorithm, and in the range of (0; 1). The R^2^ values close to 0 present the model’s poor performance, whereas the values close to 1 show good model accuracy. These values are represented by the following equations:

$$R^{2}=1-\left[\frac{\Sigma_{j=1}^{N}{\left(M_{j}-Q_{j}\right)}^{2}}{\Sigma_{i=1}^{N}{\left(M_{j}\right)}^{2}}\right]$$ (4)

$$RMSE=\sqrt{\frac{1}{N}\overset{N}{\underset{j=1}{\Sigma }}{\left(M_{j}-Q_{j}\right)}^{2}}$$ (5)

$$MAE=\frac{1}{N}\overset{N}{\underset{j=1}{\Sigma }}\left|M_{j}-Q_{j}\right|$$ (6)

where *N* is the number of samples, *M* is the target value, and *Q* is the measured value.

## 4. Methodology flow chart

In this work, the methodology in the modeling process of the shear resistance of FRP beams includes four main steps, namely:

**Step 1: Data preparation** for training the ANN model. In this step, a database of 125 experimental results is collected for the purpose of predicting the shear strength of FRP concrete beams with flexural and shear reinforcement. ANN model is built with 9 input parameters, as stated in the previous section. The data set is randomly divided into two parts: the training data set consisting of 70% of data (88 samples) and used to train the ANN model, and the testing data set consisting of the remaining 30% data (37 samples) to validate the trained ANN model.

**Step 2: Train the model**. In this step, BR algorithm is used to train and select the optimal ANN weights and bias associated with each neuron in the architecture. A training data set of 88 test samples, randomly taken from the database, were used for this purpose.

**Step 3: Model evaluation**. In this step, the trained ANN model is evaluated using the testing data set, including the remaining 37 test samples. The performance of the model is assessed by three statistical criteria, namely R^2^, RMSE, and MAE.

**Step 4: Sensitivity analysis**. This process is performed thanks to Monte Carlo simulation with the aim of evaluating the effect of each type of FRP bar on the performance of the ANN model in predicting the shear strength of FRP beams.

**Fig 3:**
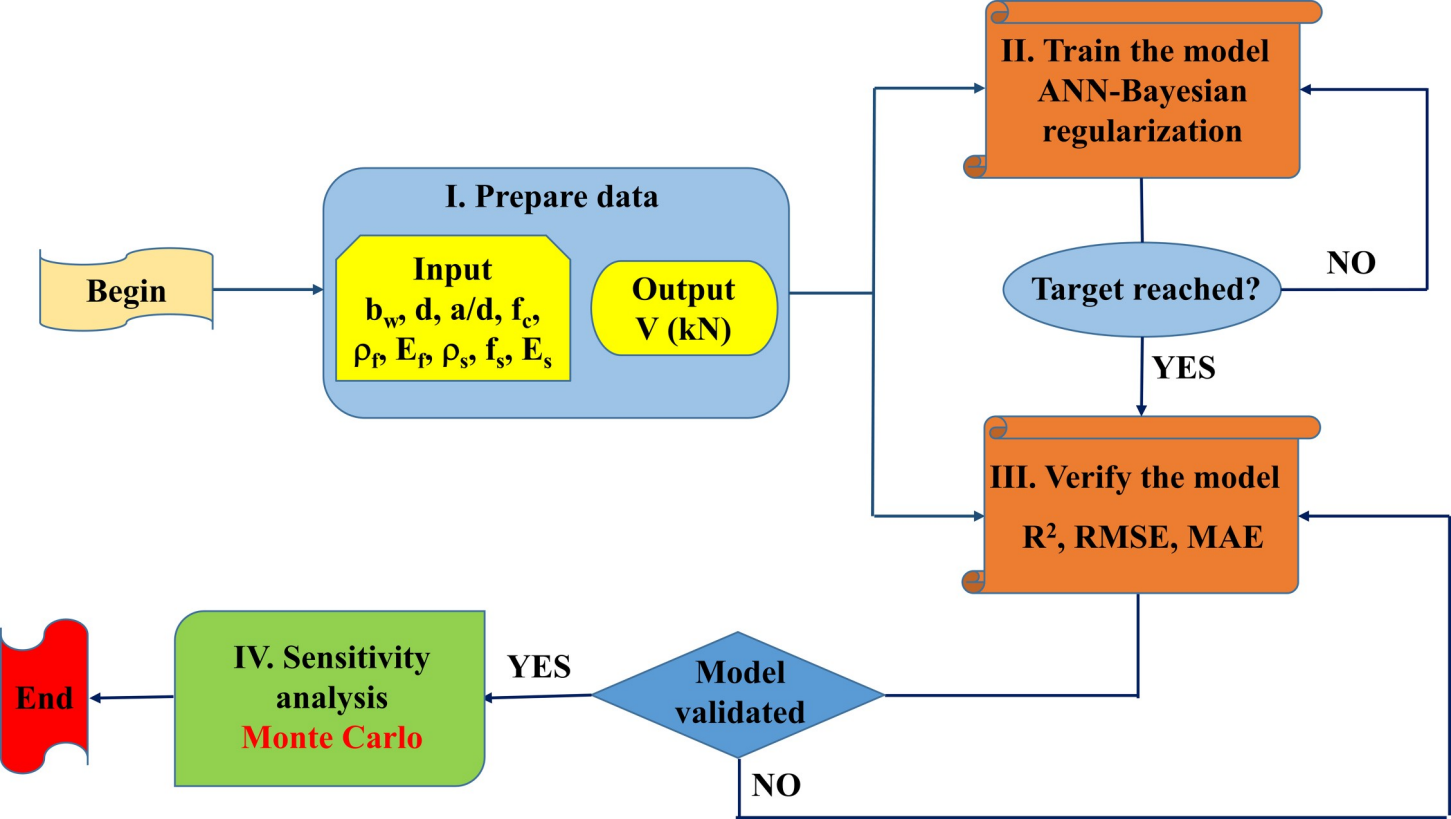
Methodology flowchart used in this study.

In summary, a schematic diagram of the methodology is shown in Fig 3.

## 5. Results & discussion

### 5.1. Investigation on the convergence of results

**Fig 4:**
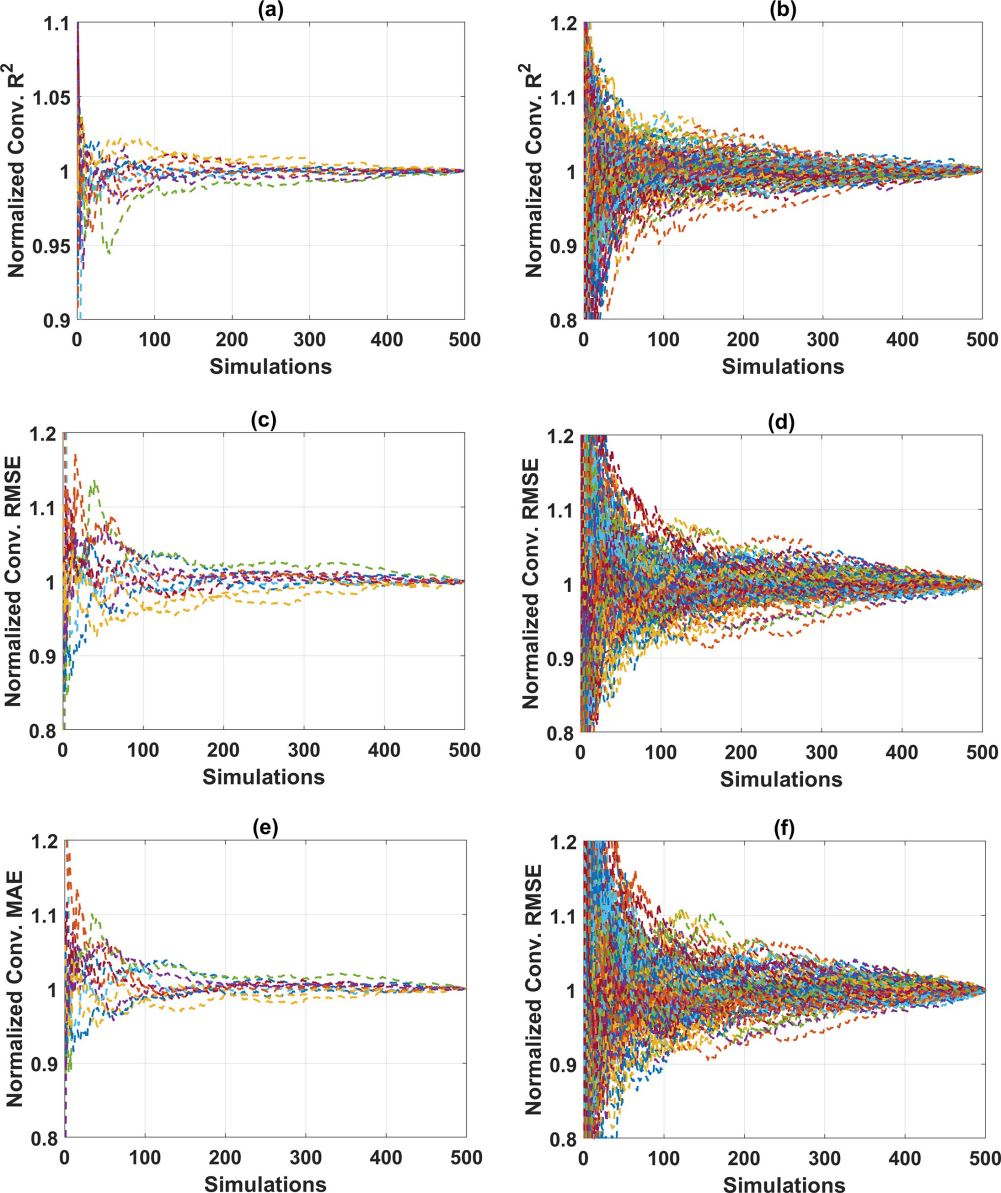
Convergence analysis for different proposed ANN architecture with respect to the testing parts: (a) R^2^ of ANN with 1 hidden layer; (b) R^2^ of ANN with 2 hidden layer; (c) RMSE of ANN with 1 hidden layer; (d) RMSE of ANN with 2 hidden layer; (e) MAE of ANN with 1 hidden layer; and (a) MAE of ANN with 2 hidden layer.

Table 4 summarizes the ANN parameters used in this study, and related information on Monte Carlo simulations. For the ANN and Monte Carlo simulation modeling, MATLAB programming language is used. Nothing that two cases of the hidden layer, consisting of 1 hidden layer and 2 hidden layers, are considered in this study. The number of neurons in each hidden layer is varied, ranging from 10 to 20 neurons, in order to cover the range of neuron as suggested in previously published works, such as in Neville (1986) [81], Gallant (1993) [82], Nagendra (1998) [83], Kannellopoulas (1997) [84]. Under the random sampling effect, the convergence of ANN model was investigated with a total of 66,000 simulations, including 121 architectures for the case of 2 hidden layers and 11 architectures for those with one hidden layer. Investigation on the convergence of results is crucial in Monte Carlo simulation, aiming at determining: (i) the appropriate number of Monte Carlo simulations, and (ii) the reliability of the simulation results. Fig 4 shows the convergence of results for all ANN architectures performed in this study. It is observed that the convergence is assured for both training, testing datasets for all cases over 500 simulations (Fig 4). Fig 4A and 4B show the normalized convergence results of R^2^. It can be seen that a fluctuation of 1% around the mean value is obtained after about 300 simulations, whereas with the same number of Monte Carlo simulations, RMSE and MAE vary about 2% of the mean values (Fig 4C–4F). Overall, the results are stable around the average values, normalized to 1 when the number of Monte Carlo simulations is 500. Thus, it can be stated that the reliable results obtained by the proposed ANN model with 1 and 2 hidden layers are converged after 500 simulations, under the random sampling effect of data. In the next step, the optimization process of different ANN architectures is performed.

**Table 4:** Summary of different ANN characteristics and investigation parameters in this study.

Parameter	Parameter	Description
Fix	Neurons in input layer	9
	Neurons in output layer	1
	Hidden layer activation function	Sigmoid
	Output layer activation function	Linear
	Cost function	Mean Square Error (MSE)
	Number of epochs	1000
	Number of simulations	500
	Training algorithm	Bayesian regularization backpropagation
Parametric study	Number of hidden layers	Varying from 1 to 2
	Neurons in hidden layer	Varying from 10 to 20, step of 1

### 5.2. Prediction performance of different ANN architectures

#### 5.2.1. ANN architectures with one hidden layer

**Fig 5:**
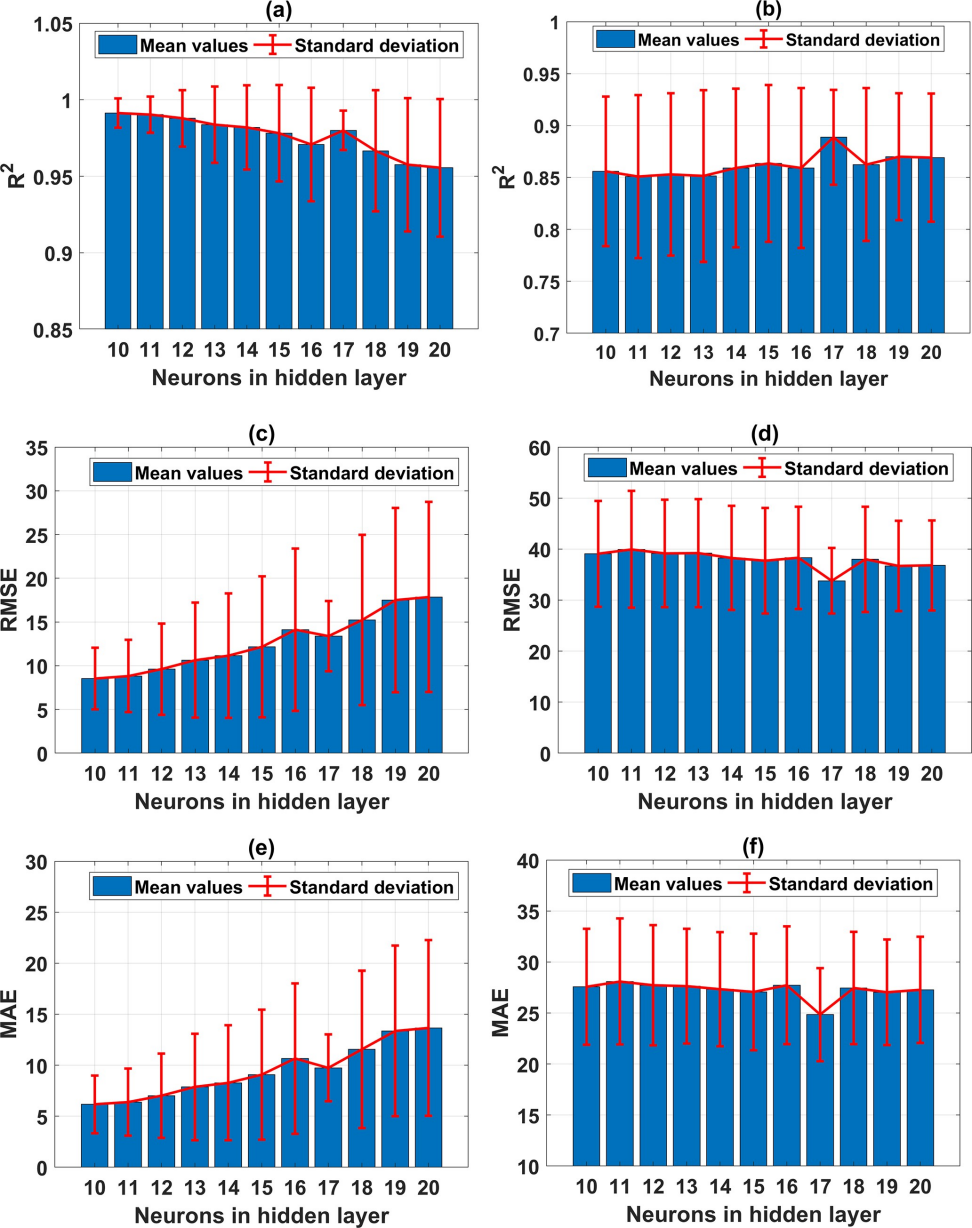
Performance of the ANN with 1 hidden layer in function of the neuron in the hidden layer, with respect to (a) mean and StD of R^2^ for the training part; (b) mean and StD of R^2^ for the testing part; (c) mean and StD of RMSE for the training part; (d) mean and StD of RMSE for the testing part; (e) mean and StD of MAE for the training part; and (f) mean and StD of MAE for the testing part.

In this section, the performance of all the ANN architectures is evaluated via statistical criteria such as the mean and standard deviation values of R^2^, RMSE, MAE. The evaluation is carried out for both the training and testing parts. Fig 5 shows the performance of ANN in function of the neuron in the hidden layer, varying from 10 to 20, regarding the mean and StD values of R^2^, RMSE, and MAE for the training and testing parts. It can be seen that the case of 17 neurons exhibits the best prediction results. In fact, the values of R^2^ are the highest compared with the other 11 cases, with the values of 0.975 and 0.8886 for the training and testing parts, respectively (cf. Fig 5A and 5B). Similarly, the values of RMSE and MAE for this architecture are also lower compared with the remaining cases, along with better standard deviation values (cf. Fig 5C–5F). Precisely, the average values of RMSE are 13.38 and 33.77 for the training, testing datasets, respectively. The average values of MAE are 9.75 and 24.84 for the training, testing datasets, respectively. It is worth noticing that the testing part’s performance is critical when evaluating the prediction capability of a model. The criteria associated with the testing dataset reflect the ability of a model to predict new data, which are not considered in the training phase of a given model. Overall, 17 neurons in the hidden layer generate the best ANN architecture with one hidden layer.

#### 5.2.2. ANN architectures containing two hidden layers

**Fig 6:**
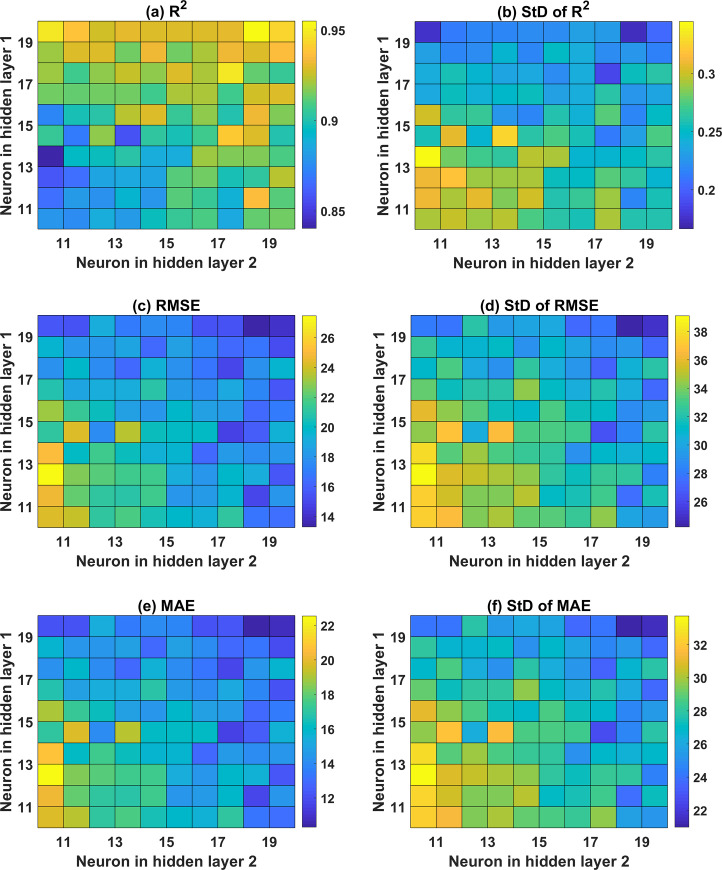
Color map of ANN with 2 hidden layers in function of the neuron in the hidden layer for the training part with respect to (a) mean values of R^2^; (b) StD of R^2^; (c) mean of RMSE; (d) StD of RMSE; (e) mean of MAE; and (f) StD of MAE.

**Fig 7:**
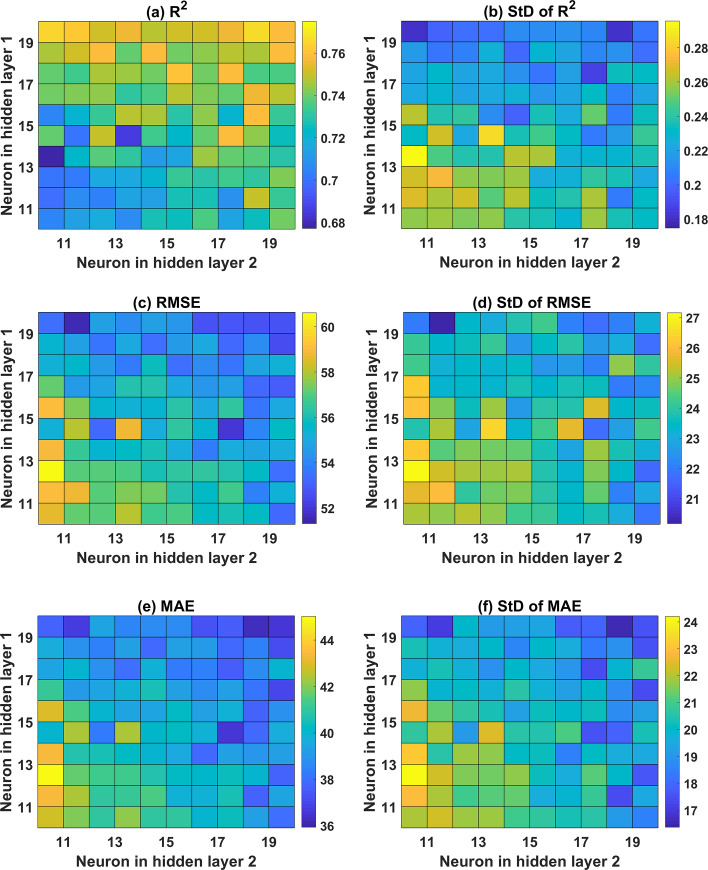
Colop-map of ANN with 2 hidden layers in function of the neuron in the hidden layer for the testing part with respect to (a) mean values of R^2^; (b) StD of R^2^; (c) mean of RMSE; (d) StD of RMSE; (e) mean of MAE; and (f) StD of MAE.

Concerning the ANN architecture with 2 hidden layers, various structures are investigated, as shown in Figs 6 and 7 for both the training and testing datasets, respectively. With respect to the training dataset, it is observed that a higher number of neurons in the second hidden layer produces higher prediction accuracy (i.e., R^2^ of 0.95, RMSE of 14, and MAE of 12). An optimal zone is observed to achieve the highest accuracy, regardless of the number of neurons in the first hidden layer. This is an excellent example to demonstrate that an appropriate ANN structure should be determined before performing any further simulations. Investigation of the effect of neurons in the two hidden layers is performed via standard deviation plots of the three error criteria (Fig 6B, 6D and 6F). The training part exhibited small standard deviation values, in general, with a higher number of neurons in both hidden layers (i.e., above 15 neurons).

Similarly, the testing part results are in good agreement with those of the training part, where the higher number of neurons in the second hidden layer is required to obtain better prediction accuracy (cf. Fig 7). However, it is clearly observed that the prediction accuracy of ANN structure with 2 hidden layers is lower than that containing 1 hidden layer. Indeed, the highest values of R^2^ are 0.7696 and 0.8886 for the cases of ANN using 2 and 1 hidden layer, respectively. Besides, the lowest values of RMSE and MAE are 51.60 and 36.50 for ANN structure using 2 hidden layers, whereas those values are 33.77 and 24.84 for ANN using 1 hidden layer, respectively.

Overall, it can be stated that ANN structure with 1 hidden layer and 17 neurons is the most effective predictor for the problem, and thus, it can be used for further investigation.

### 5.3. Prediction performance of typical ANN architecture

**Fig 8:**
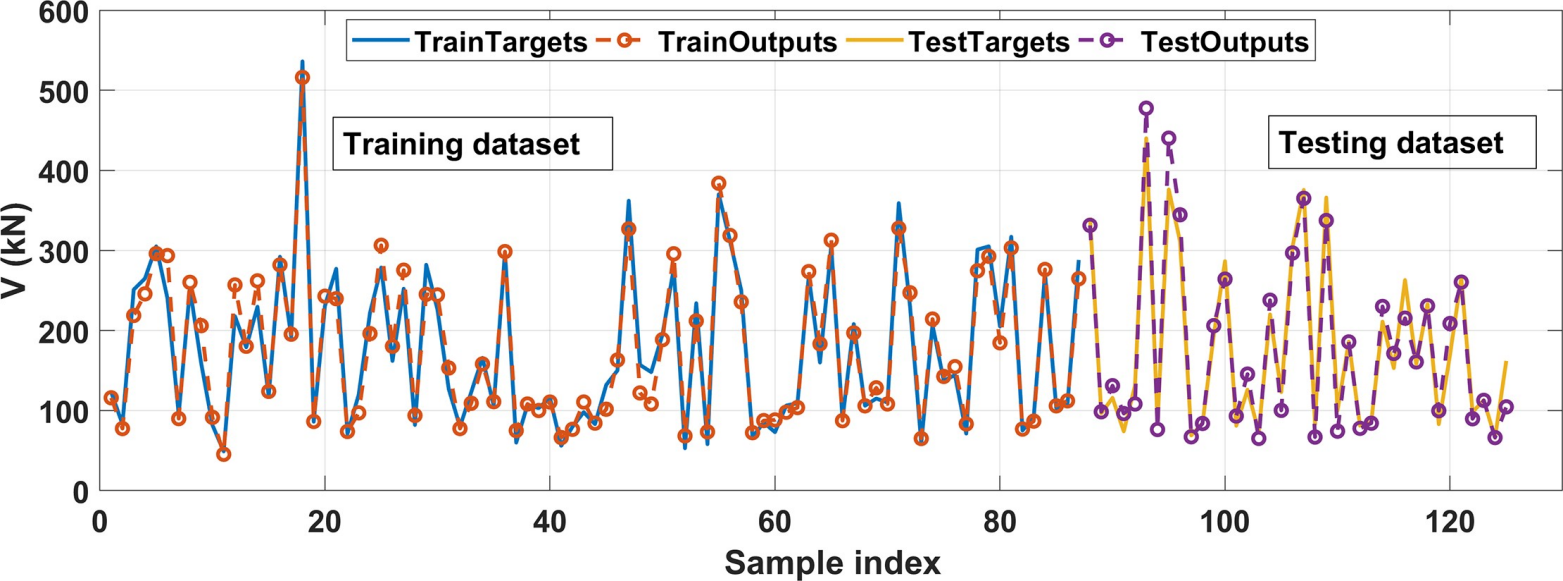
Experimental and predicted shear strength results in function of the sample index for the training and testing datasets.

**Fig 9:**
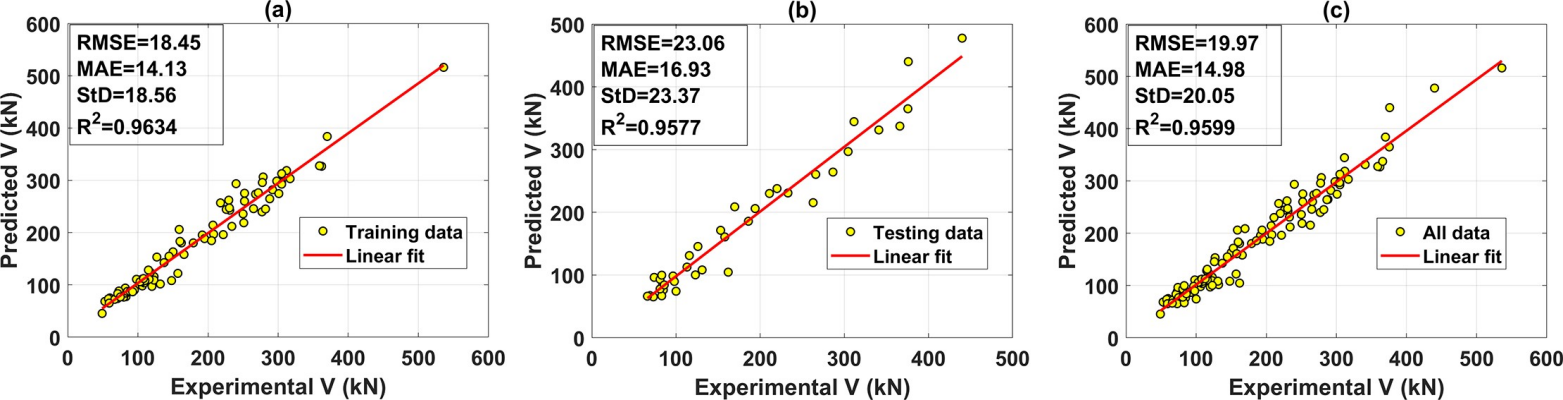
Regression graphs for the case of the best predictor ANN architecture containing 17 neurons in 1 hidden layer (a) training dataset; (b) testing dataset; and (c) all dataset.

This section is dedicated to the presentation of typical results related to the best ANN architecture containing 1 hidden layer with 17 neurons, where the R^2^ value of the testing part is highest. Fig 8 shows a comparison of experimental and predicted shear strength results in function of the sample index for the training and testing datasets. The comparison shows that the predicted shear strength values are close to the experimental ones. The predicted results of the training dataset as a function of the experimental data are shown in Fig 9A, whereas the testing part’s results are displayed in Fig 9B, respectively. The regression graph of all data is shown in Fig 9C. In these figures, the linear fits are displayed, also highlighting the R^2^, RMSE, MAE and StD values. The R^2^ values are 0.9634, 0.9577, and 0.9599 for the training, testing, and all dataset, respectively. It is observed that the linear regression lines are very close to the diagonal lines, which confirms the strong correlation between predicted and experimental shear strength. In terms of RMSE, MAE, and StD values, the best ANN architecture gives a high prediction performance. The RMSE values are 18.45 MPa, 23.06 MPa, and 19.97MPa; MAE values are 14.13 MPa, 16.93 MPa, and 14.98 MPa; StD values are 18.56, 23.37, and 20.05 for the training, testing, and all data, respectively. A good agreement between the predicted and the experimental shear strength of FRP concrete beam is obtained

Finally, several previously published models and the corresponding accuracy are given for comparison purposes (cf. Table 5). It could be seen that the proposed ANN model in this study exhibits good accuracy compared with other works. The accuracy of the best model (R^2^ = 0.960) is slightly inferior to the accuracy obtained by the Multivariate adaptive regression splines (MARS) model in the work of Abbasloo et al. [54] (R^2^ = 0.974). However, the authors used a smaller amount of samples (112 data) than this work (125 data). This might be the reason for a small difference in prediction accuracy. Second, the present study uses a smaller number of inputs (9 inputs) than in the work of Abbasloo et al. [54] (12 inputs). Without affecting too much the prediction accuracy, the reduction of 3 inputs is not significantly pronounced with a small dataset, but might be of great interest while considering a bigger dataset or industrial design context.

**Table 5:** Comparison with the literature for prediction of shear strength of fiber reinforcement bars concrete beams.

Ref.	Machine learning algorithm	Inputs	Sample size	Performance measure
Nehdi et al. [2]	Genetic algorithm	6 inputs: the beam’s effective depth (d) and with (b_w_); the shear span to depth ratio (a/d); the longitudinal reinforcement ratio (ρ_fl_); the compressive strength of concrete (f’_c_); the ratio of the modulus of elasticity of FRP to that of steel (E_fl_/E_s_); the ultimate capacity of FRP shear reinforcement (f_fυ_).	168 data include: 100 data with shear reinforcement, 68 data without shear reinforcement	AAE = 22.42% V_m_ = V_measured_ V_cal_ = V_calculated_ $AAE=\frac{1}{n}\Sigma \frac{\left|V_{m}-V_{cal}\right|}{V_{m}}\times 100$
Abbasloo et al. [54]	Multivariate adaptive regression splines (MARS) - M5’	12 inputs: the average concrete compressive strength (f_cm_); the ratio of shear span to the effective depth (a/d); the cross-section width (b); the effective depth of the cross-section (d); the area of longitudinal and transversal reinforcement (A_F_ and A_Fw_); the ultimate tensile strength of longitudinal and transversal reinforcement (f_Fu_ and f_Fwu_); Young’s modulus of longitudinal and transversal reinforcement (E_F_ and E_Fw_); the longitudinal and transversal reinforcement ratio (ρ_F_ and ρ_Fw_).	112 data with shear reinforcement	R^2^ = 0.9735 (MARS) RMSE = 9.03 (MARS) R^2^ = 0.9004 (M5’) RMSE = 32.52 (M5’)
This study	ANN—Bayesian Regularization (BR)	9 input: Beam width (bw); Effective depth (d); The ratio of the shear span to the effective depth (a/d); Compressive strength of concrete (fc); Longitudinal FRP reinforcement ratio (ρ_f_); Modulus of elasticity of longitudinal FRP reinforcement (E_f_); FRP shear reinforcement ratio (ρ_s_); Tensile strength of FRP shear reinforcement (f_s_); Modulus of elasticity of FRP shear reinforcement(E_s_).	125 data with shear reinforcement	**Training data** R^2^ = 0.9634 RMSE = 18.45 MAE = 14.13 **Testing data** R^2^ = 0.9577 RMSE = 23.06 MAE = 16.93 **All data** R^2^ = 0.9599 RMSE = 19.97 MAE = 14.98

### 5.4. Analysis of prediction accuracy based on reinforcement type

**Fig 10:**
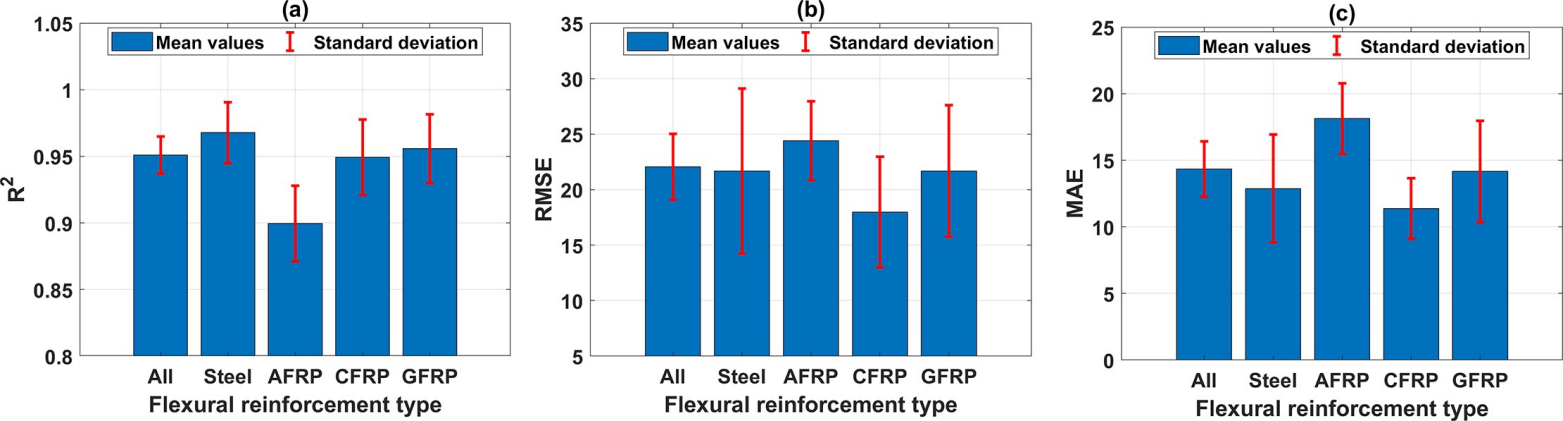
Performance of the best ANN architecture containing 17 neurons with 1 hidden layer in function of flexural reinforcement type, with respect to (a) mean and StD values of R^2^; (b) mean and StD values of RMSE; and (c) mean and StD values of MAE.

In this section, the prediction capability of the best ANN architecture (i.e., ANN-9-17-1) is analyzed considering different types of FRP reinforcements. The performance of the best ANN architecture in function of flexural reinforcement type is investigated by mean and StD values of R^2^ (Fig 10A), mean and StD values of RMSE (Fig 10B), and mean and StD values of MAE (Fig 10C) for 500 Monte Carlo simulations. In fact, the accuracy of ANN model in predicting the shear strength is strongly dependent on the types of flexural reinforcement, namely steel, AFRP, CFRP, and GFRP. It is observed that the shear strength prediction by the proposed ANN architecture for AFRP has the lowest accuracy comparing with other types. In this case, the mean value of R^2^ is the lowest (i.e., 0.90), and those of RMSE (i.e., 24.40), MAE (i.e., 18.13) are the highest. Besides, the prediction accuracy remains similar compared with other cases of flexural reinforcement. It is worth noticing that the prediction accuracy of ANN for AFRP type flexural reinforcement is the lowest, even though the AFRP flexural reinforcement type possesses the highest number of samples (41 samples as indicated in Table 3). This might come from the possible interaction between both types of reinforcement (flexural and shear), or an inappropriate range of inputs and output compared with those of the full dataset. Thus, further investigation of this behavior should be considered.

**Fig 11:**
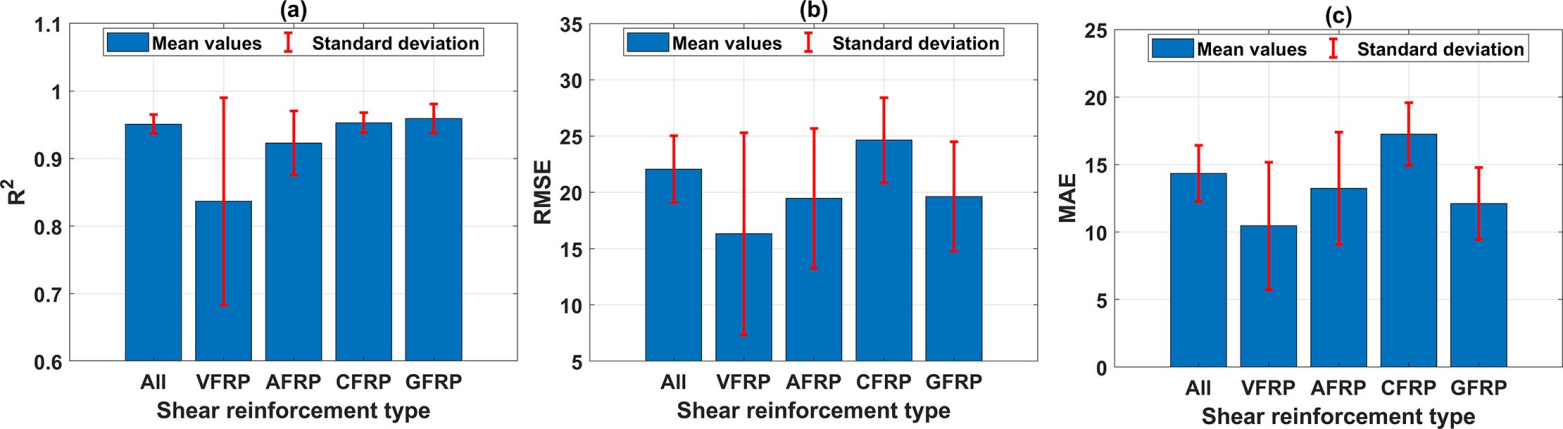
Performance of the best ANN architecture containing 17 neurons with 1 hidden layer in function of shear reinforcement type, with respect to: (a) mean and StD of R^2^; (b) mean and StD of RMSE; (c) mean and StD of MAE.

Fig 11 shows the performance of the best ANN architecture containing 17 neurons with 1 hidden layer in function of shear reinforcement type. Similar to the previous investigation, four types of reinforcement, including VFRP, AFRP, CFRP, and GFRP, significantly influence the ANN model accuracy in predicting the shear strength. The mean value of R^2^ in predicting the shear strength for the case of VFRP is the lowest (Fig 11A). However, the mean values of RMSE and MAE are the smallest (cf. Fig 11B and 11C), and the StD values, in this case, are significantly higher compared with other types of shear reinforcement. It could be concluded that the proposed ANN model is sensitive in predicting such type of reinforcement, which might come from the insufficient number of samples (only 10 samples, see Table 3). Moreover, the mean value of R^2^ in predicting the shear strength using AFRP, CFRP, and GFRP shear reinforcement is higher than that of VFRP, but the mean values are higher than those with AFRP, CFRP, and GFRP. In this case, it is difficult to evaluate the model’s accuracy based on the mean values of these statistical measurements. However, based on the StD values of R^2^, RMSE, and MAE, it could be stated that the model accuracy is very sensitive in predicting VFRP as shear reinforcement.

## 6. Conclusion

In spite of numerous investigations on the shear strength prediction of FRP concrete beam, the performance of prediction could still be enhanced from more in-depth investigations. In this paper, the current investigation displays a simple but efficient model to use an ANN architecture for predicting the shear strength of FRP concrete beam with both flexural and shear reinforcements. Two cases of the hidden layer number containing various numbers of neuron are proposed to find out the best architecture of ANN model. After the performance analysis of different architectures, the best architecture of ANN model is proposed, where the robustness is supported by the presence of a random dataset splitting over 500 Monte Carlo simulations. Statistical results assessment of Monte Carlo simulations is derived to validate the prediction reliability of results and evaluate the ANN model’s convergence. A simple and ANN architecture containing 17 neurons in 1 hidden layer is proven to predict the shear strength of FRP concrete beam with excellent agreement between model and experimental results, where the highest value of R^2^ could reach R^2^ = 0.9599 for all data. The sensitivity analysis of the accuracy of the best ANN architecture is next investigated in function of different types of flexural and shear reinforcement. The ANN model of the present investigation could facilitate and improve the use of ANN model in different problems relating to civil engineering.

## Supporting information

S1 Data (XLSX)
